# Case Report: Three-year follow-up of TACE for recurrent undifferentiated pleomorphic sarcoma: sustained local control and no systemic progression

**DOI:** 10.3389/fonc.2025.1631000

**Published:** 2025-08-05

**Authors:** Xiangrui Chen, Min Hu, Chengluo Hao, Limei Ma, Yunwei Han

**Affiliations:** ^1^ Department of Oncology, Zigong Third People’s Hospital, Zigong, Sichuan, China; ^2^ Department of Oncology, Affiliated Hospital of Southwest Medical University, Luzhou, Sichuan, China; ^3^ Department of Dermatology, The Third People’s Hospital of Zigong, Zigong, Sichuan, China; ^4^ Pathology Department, The Third People’s Hospital of Zigong, Zigong, Sichuan, China

**Keywords:** transcatheter arterial chemoembolization (TACE) therapy, recurrent undifferentiated pleomorphic sarcoma (UPS), tumor vascular embolization, long-term follow-up, tumor control

## Abstract

We report a case of recurrent undifferentiated pleomorphic sarcoma (UPS) in the right calf of a 48-year-old female patient who had undergone initial surgical resection in February 2018. The patient underwent two sessions of transcatheter arterial chemoembolization (TACE) between June and July 2022. During the procedures, superselective catheterization of the tumor-feeding arteries via the posterior tibial artery was performed, followed by infusion of epirubicin and sequential embolization with lipiodol, 300-500 μm microspheres, and gelatin sponges. Post-procedural angiography confirmed complete vascular occlusion. Clinical and imaging follow-up demonstrated significant tumor necrosis (60-70%) at one month post-treatment with progressive tumor shrinkage and central liquefaction. The disease remained stable at six months without distant metastasis. While literature reports of TACE for advanced sarcomas show PFS of 6.3–21 months, our case achieved 36-month PFS in locally recurrent non-metastatic UPS—suggesting greater efficacy in earlier disease stages. During 36 months of follow-up, sustained local control was achieved without systemic progression. These findings suggest that for vascular-rich recurrent UPS when surgical resection is challenging, TACE represents a feasible locoregional therapeutic option.

## 1 Introduction

Undifferentiated pleomorphic sarcoma (UPS), as an aggressive soft tissue sarcoma (1–3), presents significant clinical management challenges when it recurs locally. While radical surgical resection remains the cornerstone of initial treatment, repeat surgery often proves unfeasible due to anatomical constraints from prior operations or neurovascular involvement (4). Furthermore, systemic chemotherapy and radiation therapy typically yield suboptimal outcomes for recurrent lesions, underscoring the urgent need for effective alternative locoregional therapies (5).

Although transarterial chemoembolization (TACE) has well-established efficacy in hypervascular malignancies such as hepatocellular carcinoma (6), its application in extrahepatic sarcomas like UPS remains understudied. Existing limited evidence—primarily from retrospective studies and case reports of other sarcoma subtypes like angiosarcoma—suggests potential tumor response with TACE (7). However, published data specifically addressing TACE’s efficacy in hypervascular recurrent UPS of the limbs—particularly regarding long-term limb salvage and functional outcomes—remain exceptionally scarce. This discrepancy likely reflects our patient’s favorable disease biology (local recurrence without metastasis) versus their cohorts with systemic disease. The technical efficacy observed here—complete devascularization—echoes Hansch’s report of successful UPS preoperative embolization.

To address this gap, we present a case of palliative TACE for recurrent UPS in the right calf. Our approach involved superselective catheterization of tumor-feeding arteries, with epirubicin infusion and sequential embolization using lipiodol-microspheres-gelatin sponge. We document this strategy achieving 36-month local tumor control and functional limb preservation in a patient unsuitable for repeat surgery who declined chemoradiation.

## 2 Case description

All procedures involving human participants in this study were conducted in strict accordance with the ethical standards of the Ethics Committee of Zigong Third People’s Hospital and with the 2013 World Medical Association Declaration of Helsinki. Written informed consent was obtained from the patient for the publication and scientific use of this case report, including anonymized imaging data and clinical details. Measures were taken to ensure that no personally identifiable information (e.g., facial features, full-name identifiers) could be disclosed, thereby safeguarding patient privacy in compliance with local and international regulations.

### 2.1 Clinical manifestations and initial diagnosis

**Figure 1:**
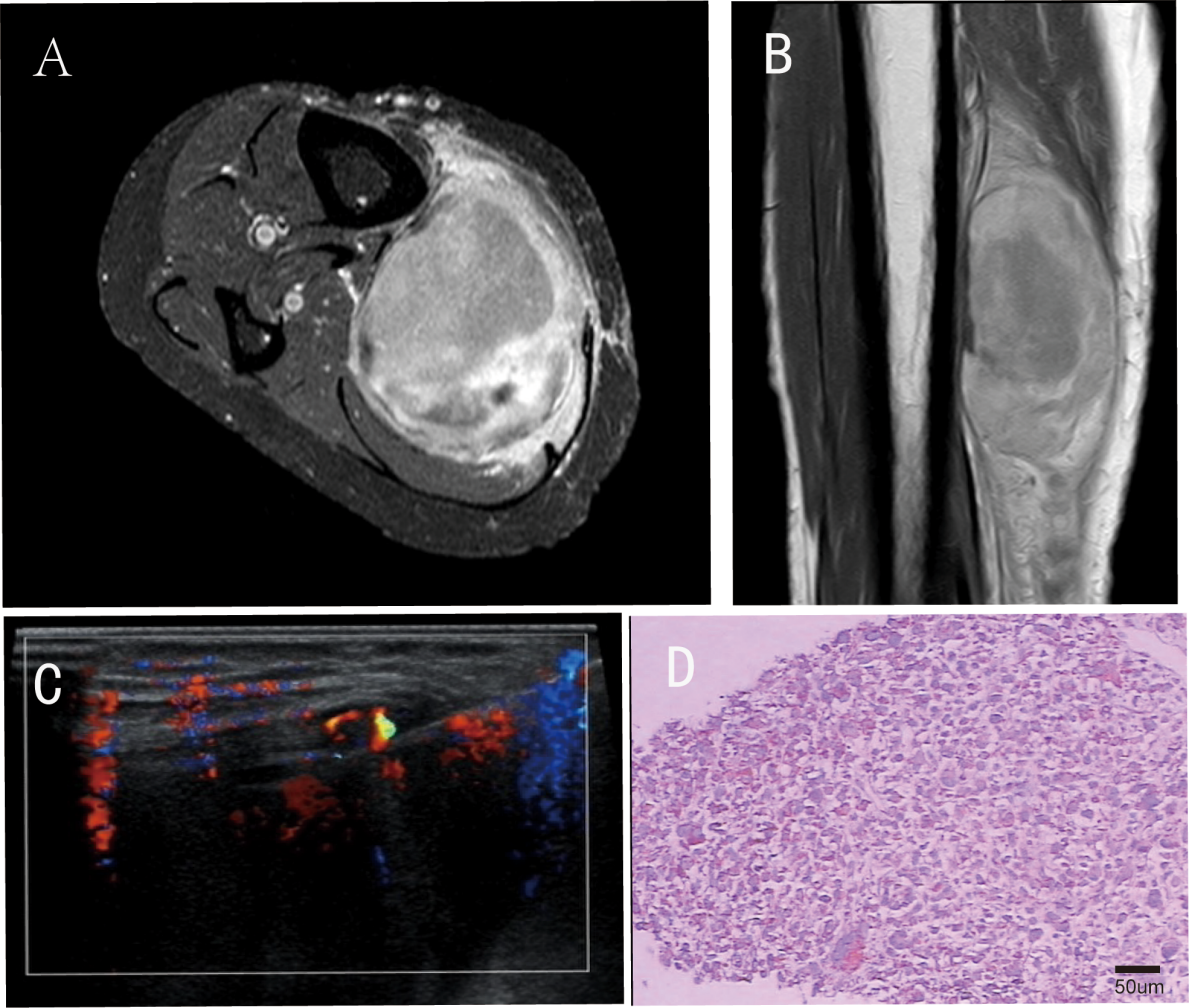
Imaging and pathological conditions of patients before surgical operation. In the panel **(A)** shows the horizontal section of the preoperative magnetic resonance (MR) T2-weighted image of the patient; **(B)** presents the coronal section of the preoperative magnetic resonance (MR) T2-weighted image; **(C)** represents the ultrasound-guided percutaneous biopsy; **(D)** Postoperative histopathology (H&E staining, original magnification ×200; scale bar = 50μm).

A 48-year-old female patient presented to the orthopedic outpatient clinic in January 2018 with a painless, progressively enlarging mass in the right lower leg. Magnetic resonance (MR) imaging revealed a hyperintense lesion in the right lower leg on T2-weighted sequences, while computed tomography (CT) of the right leg showed a low-density mass. Staging examinations, including chest/abdomen/pelvis CT, did not detect any distant metastases. Subsequently, an ultrasound-guided core needle biopsy was performed, and histopathological examination confirmed the diagnosis of malignant fibrous histiocytoma (**Figure 1**). Immunohistochemical staining results: Vimentin (++), CD68 (focal +), Myoglobin (-), Desmin (-), H-caldesmon (-), CD34 (-), S-100 protein (-), NSE (-), Ki-67 index: approximately 1% positive cells. The clinical intervention timeline for this recurrent undifferentiated pleomorphic sarcoma is detailed in **Table 1**.

**Table 1:** Timeline of clinical interventions.

Time Point	Clinical Intervention/Procedure	Key Technical Details
Jan 2018	Initial diagnosis	Ultrasound-guided biopsy of right calf mass
Feb 2018	Tumor resection	Wide local excision with R0 margins
Jun 2022	Recurrence confirmation	CT/MRI-guided biopsy confirming UPS
Jun 13, 2022	First TACE session	Epirubicin 50mg + Lipiodol + 300-500μm microspheres + Gelfoam
Jul 14, 2022	Second TACE session	Epirubicin 50mg + 100-300μm microspheres
Aug 2022	1-month clinical follow-up	Physical examination + functional assessment
Jan 2023	6-month imaging surveillance	CT: stable tumor
Apr 2025	Final follow-up visit	Clinical + MRI evaluation

Chronology of key interventions for recurrent UPS. TACE, transcatheter arterial chemoembolization; UPS, undifferentiated pleomorphic sarcoma; R0, microscopically negative resection margins.

In February 2018, the patient underwent tumor resection surgery. Postoperative histopathological examination confirmed complete tumor excision with negative margins.

### 2.2 Tumor recurrence

**Figure 2:**
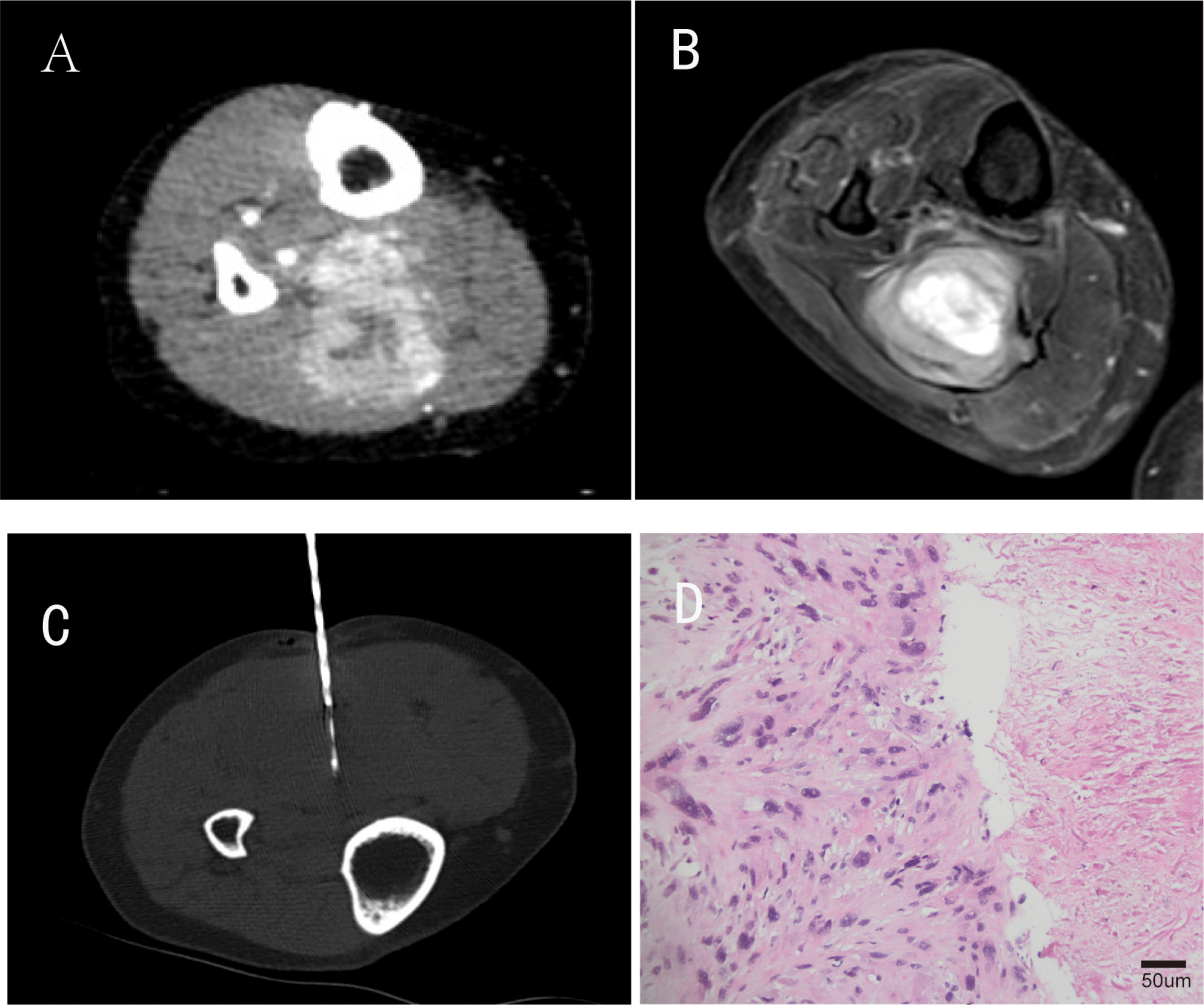
Partial imaging and pathological findings of tumor recurrence. **(A)** is an enhanced CT image during the arterial phase of the patient’s tumor recurrence four years after surgical resection; **(B)** is a coronal T2-weighted magnetic resonance imaging (MRI) sequence; **(C)** represents CT-guided percutaneous biopsy performed after tumor recurrence; **(D)** Repeat biopsy histopathology(H&E staining, original magnification ×200; scale bar = 50μm).

The patient remained clinically stable for approximately 4 years post-surgery until June 2022, when she presented with a painful mass at the original surgical site. Pre-treatment assessment revealed moderate pain (Visual Analog Scale [VAS] score: 6/10) and impaired limb function (Musculoskeletal Tumor Society 1993 scoring system [MSTS-93] score: 18/30), primarily due to tumor-related mechanical compression and perineural irritation. Magnetic resonance imaging (MRI) and contrast-enhanced computed tomography (CT) confirmed local recurrence, with the tumor measuring approximately 65 × 52 mm. Staging CT scans of the chest/abdomen/pelvis did not reveal any distant metastases. A repeat CT-guided biopsy was performed, and histopathological examination of the biopsy specimens suggested a malignant spindle cell tumor, most likely pleomorphic sarcoma (**Figure 2**).

The decision to proceed with TACE rather than alternative therapies for this recurrent UPS was made by the multidisciplinary team (MDT) based on three key factors: (1) imaging-confirmed tumor biology suitability (DSA/MRI-demonstrated >120% enhancement with ≥3 independent feeding arteries, fulfilling TACE response predictors, while peroneal vascular invasion and post-surgical anatomical distortion precluded reoperation), (2) the patient’s explicit refusal of radiotherapy/chemotherapy, and (3) the lesion’s complex vascular characteristics that favored transarterial intervention over other modalities.

### 2.3 Transcatheter arterial chemoembolization

Two sessions of palliative TACE were performed as a locoregional salvage therapy for recurrent UPS, representing an off-label but biologically justified approach given the tumor’s hypervascularity.

First TACE Procedure (June 13, 2022): Using the Seldinger technique, the right femoral artery was accessed in a retrograde manner, and a 5F Cobra catheter was placed in the right femoral artery. Selective angiography revealed three independent tumor-feeding arteries branching from the posterior tibial artery. The tumor exhibited dense, hyperenhancing “egg-shaped” vascular staining in the early arterial phase, with thickened and tortuous tumor-supplying vessels. A microcatheter was used to perform superselective catheterization of the tumor-feeding arteries. Epirubicin (50 mg) was infused through the feeding arteries, followed by embolization with lipiodol and 300–500 µm blank microspheres. Further embolization was achieved using gelatin sponge particles, resulting in complete vascular occlusion. Postprocedural angiography confirmed complete tumor devascularization.

**Figure 3:**
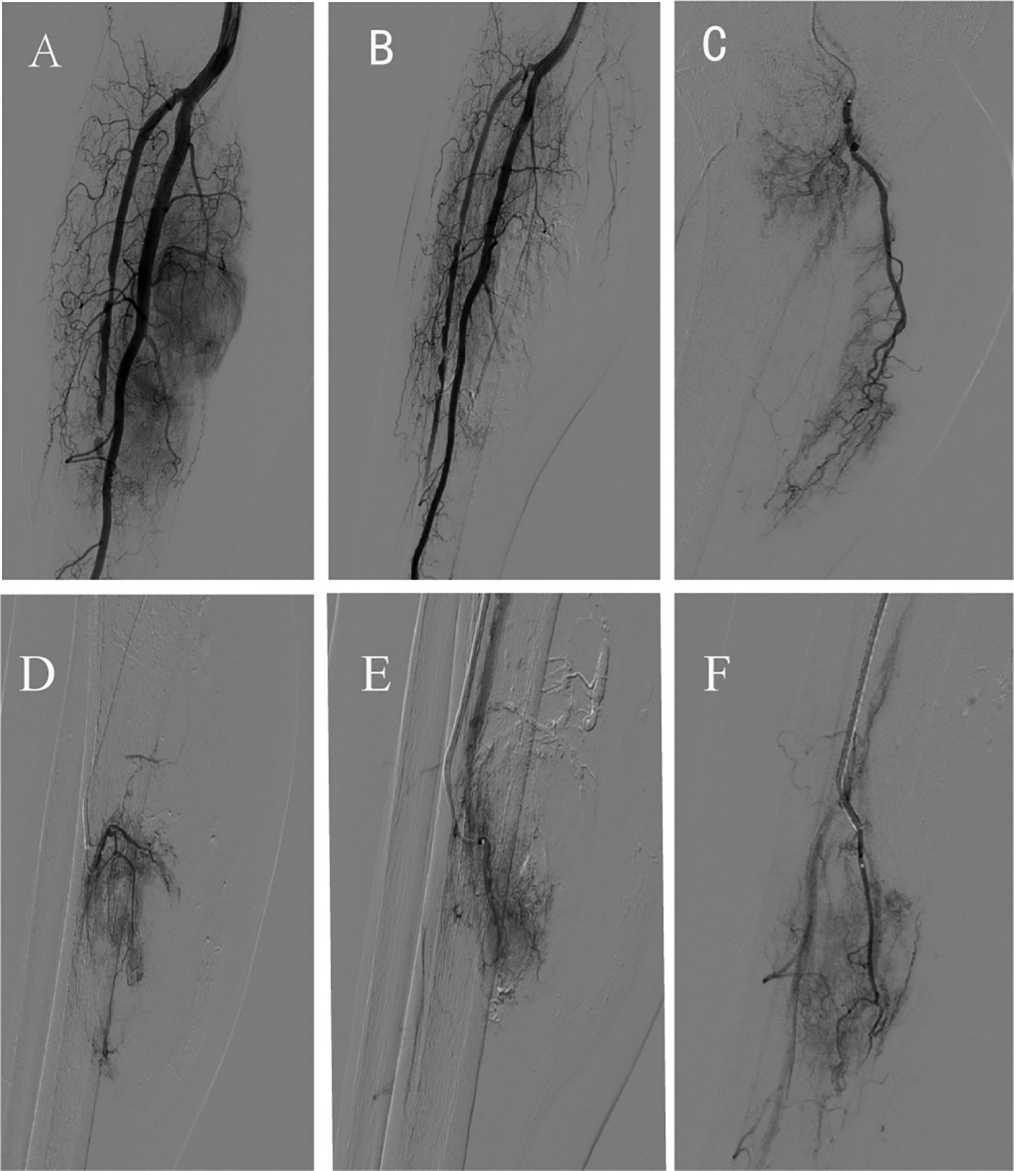
Imaging of the second transcatheter arterial chemoembolization. **(A)** Pre-procedure angiography; **(B)** Post-procedure angiography after transcatheter arterial chemoembolization; **(C-F)** Superselective angiography of partial tumor-feeding arteries.

Second TACE Procedure (July 14, 2022): Angiography showed four independent tumor-feeding arteries branching from the posterior tibial artery, with evidence of small collateral tumor-supplying vessels having reformed. Using a similar approach, all four tumor-feeding arteries were superselectively catheterized. Epirubicin (50 mg) was infused, followed by embolization with 100–300 µm microspheres and larger particles, ultimately achieving complete vascular cutoff (**Figure 3**).

### 2.4 Follow-up

One month after the second TACE procedure, the patient’s symptoms (pain and swelling) significantly improved, enabling independent ambulation without assistive devices (pre-treatment pain score: 6/10 on VAS; post-TACE: 2/10). Contrast-enhanced MRI follow-up showed marked tumor shrinkage, with central tumor necrosis and iodine deposition. A repeat biopsy of three tissue samples confirmed extensive tumor necrosis (60–70%) with fibrous tissue proliferation.

**Figure 4:**
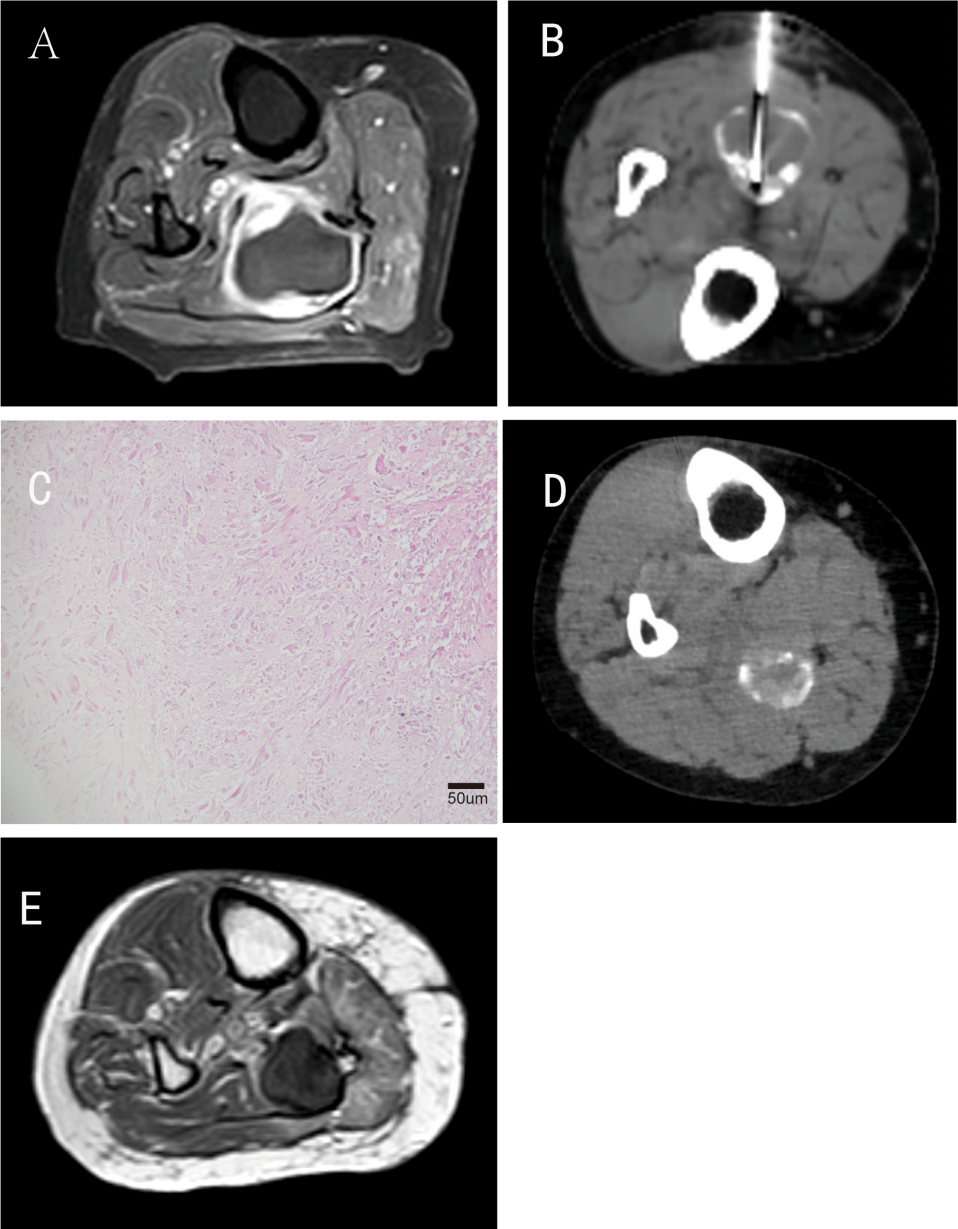
Follow-up of the patient after interventional therapy​. **(A)** Contrast-enhanced T1-weighted MRI performed 1 month after the second interventional procedure; **(B)** Follow-up CT scan and CT-guided repeat biopsy conducted 1 month after the second interventional procedure; **(C)** Pathology revealed extensive tumor necrosis (60%–70%) with fibrous tissue proliferation (H&E staining, original magnification ×200; scale bar = 50μm); **(D)** CT performed 6 months postoperatively showed partial reduction of iodized oil deposition; **(E)** MR performed 35 months postoperatively indicated a local residual soft-tissue lesion without significant enhancement.

Six months after the second TACE procedure, the patient returned for follow-up, and a CT scan of the right lower leg showed tumor stability. Functional assessment revealed full independence in activities of daily living (ADLs), including walking >1000 meters without claudication, climbing stairs, and self-care; MSTS-93 limb function score reached 28/30, indicating excellent local functional recovery. By April 2025, the patient remained clinically stable, with no evidence of systemic metastases on follow-up chest/abdomen CT scans (**Table 2**). A follow-up MRI of the right lower leg revealed a small residual soft-tissue lesion without significant enhancement, indicating persistent tumor control (**Figure 4**).

**Table 2:** Therapeutic outcomes and corresponding findings.

Time Point	Imaging Findings	Pathological/Functional Outcomes	Figure Reference
Jan 2018	T2WI hyperintense lesion (65×52 mm)	Biopsy: UPS confirmed	**Figures 1A–D**
Jun 2022	Contrast CT: Hypervascular tumor (43×31 mm, enhancement >120%)	VAS: 6/10; MSTS-93: 14/30, [RECIST Baseline]​	**Figures 2A–D**
Jun 13, 2022	DSA: 3 feeding arteries	No adverse events	–
Jul 14, 2022	DSA: Complete devascularization	No adverse events, Symptom relief (VAS 6→2)	**Figure 3B**
Aug 2022	MRI: Tumor shrinkage (39×27 mm) + lipiodol deposition, RECIST: SD	MSTS-93: 28/30; Necrosis confirmed (biopsy)	**Figure 4A**
Jan 2023	CT: Stable tumor (22×19 mm) + reduced lipiodol, RECIST: PR	Pain-free; Independent ADL	**Figure 4D**
Apr 2025	MRI: Stable tumor (20×18 mm) +Non-enhancing residual, RECIST: PR	Asymptomatic; MSTS-93: 28/30	**Figure 4E**

Longitudinal outcomes after interventions. VAS, Visual Analog Scale; MSTS-93, Musculoskeletal Tumor Society 1993 scoring system; DSA, digital subtraction angiography; ADL, activities of daily living. Necrosis rate quantified via biopsy. RECIST 1.1 criteria: SD = stable disease (<30% reduction); PR = partial response (≥30% reduction). Adverse events assessed by CTCAE v5.0.

Notably, over the 3-year follow-up period, the patient reported sustained absence of pain (VAS=0), limb edema, or functional impairment, maintaining complete autonomy in daily life and occupational activities. Although biannual surveillance was recommended per institutional protocol, the patient attended irregular visits (last visit: April 2025) due to asymptomatic status and preserved quality of life—a pattern consistent with favorable responders in limb sarcoma interventions.

## 3 Discussion

The treatment of recurrent UPS in the extremities is challenging due to its highly vascularized structure, aggressive local invasiveness, and limited response to radiotherapy or systemic therapy (1, 8). From a biological perspective, UPS and MFS exhibit complex molecular profiles characterized by genomic instability, often diagnosed in the extremities or trunk of elderly patients (9). This molecular complexity contributes to tumor heterogeneity, further complicating treatment (10).

Systemic chemotherapy remains the primary approach for locally advanced and metastatic UPS (5). Although anthracycline-based regimens are the standard of care, the prognosis for advanced or metastatic UPS remains poor (8). Recent studies suggest that UPS may exhibit unique sensitivity to ICI due to its distinct biological features (11). Multiplatform analyses have identified potential biomarkers and novel therapeutic targets (12), providing a foundation for personalized treatment. However, the clinical validation and application of these biomarkers remain challenging, requiring further research to confirm their accuracy and reliability across diverse patient populations (13). The recent documentation of co-occurring KRAS G12D and TP53 R249S mutations in a chemo-refractory hepatic UPS (14)- matching our patient’s epirubicin use - highlights profound molecular heterogeneity. This evidence reinforces the need to explore TACE synergies with mutation-targeted agents in resistant cases.

TACE, a well-established technique for liver malignancies, has been increasingly applied in sarcomas and other hypervascular tumors (15–17). Evidence from clinical studies indicates that TACE may serve as a salvage treatment for recurrent sarcomatoid hepatocellular carcinoma post-resection (18); however, its efficacy is limited in advanced cases with portal vein tumor thrombosis and systemic metastasis (e.g., stage T4 SHC), potentially leading to treatment resistance and rapid progression (as demonstrated by hepatic extravasation occurring within 47 days postoperatively in this case). For giant malignant phyllodes tumors (>20 cm in maximum diameter) containing angiosarcoma components, preoperative sequential TACE (4 cycles of epirubicin with embolization microspheres) can achieve significant tumor volume reduction (45%), enabling radical surgery with chest wall preservation while avoiding complex skin grafting reconstruction (19). For chemotherapy-resistant hepatic metastases from retroperitoneal leiomyosarcoma, drug-eluting bead TACE combined with anlotinib targeted therapy demonstrated prolonged disease control (progression-free survival reaching 29 months), suggesting a potential transformative therapeutic approach for advanced sarcomas (20). In this case, superselective catheterization and lipiodol emulsion enabled precise drug delivery and vascular occlusion. The mechanism of TACE involves not only localized high-dose chemotherapy but also tumor ischemia via vascular embolization, inhibiting growth and metastasis (6). Studies indicate that while TACE demonstrated tumor reduction in this hypervascular case (notably, our 60-70% necrosis rate aligns with the 30-70% objective responses in advanced sarcoma TACE studies (21, 22), while the sustained 36-month progression-free survival far exceeds their reported medians of 6.3–21 months), its efficacy may be limited in hypovascular subtypes, alleviate symptoms, and improve quality of life in hypervascular STS (7). However, its efficacy depends on tumor vascularity and biological behavior, with collateral circulation potentially limiting its effectiveness.

Sarcomas of the limbs/trunk (e.g., UPS) demonstrate higher R0 resection rates (negative margins) and superior 5-year survival due to superficial anatomical locations and well-defined boundaries (23). However, local recurrence rates for limb UPS remain >30%, primarily attributable to microscopic residual disease at tumor margins or neurovascular bundle infiltration (24). Secondary wide resection is often compromised by functional preservation requirements, while radiotherapy efficacy for recurrences is limited by cumulative dose constraints and tissue fibrosis risks. Conversely, abdominal/retroperitoneal sarcomas are frequently diagnosed at advanced stages (tumor size >10 cm) with proximity to vital organs/vasculature (e.g., retroperitoneal UPS involving renal vessels), resulting in R0 resection rates below 50%. Extended resections requiring multivisceral excision increase complication risks by 30%, with minimal survival benefit from adjuvant chemoradiation. Retroperitoneal sarcomas exhibit >20% lower 5-year survival than limb tumors, higher distant metastasis rates, and unique biological behaviors (e.g., VEGF overexpression, hypervascularity) potentially driving aggressive progression. Addressing therapeutic challenges in recurrent limb UPS, this study explores interventional embolization as an alternative strategy: superselective embolization of tumor-feeding arteries induced 60-70% tumor necrosis while ablating collateral circulation. Post-TACE functional recovery (MSTS-93 score: 18→28/30) and 36-month progression-free survival suggest potential for durable local control in hypervascular recurrences. While this single case cannot establish a new therapeutic paradigm, it contributes preliminary evidence for TACE as a limb-preserving option in select patients ineligible for reoperation or radiotherapy.

This study acknowledges inherent limitations as a single-case report. Our findings remain anecdotal and require validation in larger cohorts. Tumor vascular heterogeneity remains a key predictor of TACE efficacy, with current findings potentially applicable only to hypervascular UPS subtypes. Significant genetic heterogeneity may drive differential treatment responses, while comorbidities could alter drug metabolism. To address the scarcity of clinical TACE data for UPS, preclinical validation is imperative: orthotopic murine models with patient-derived xenografts can simulate human vascular heterogeneity, and novel embolic agents have demonstrated efficacy in eliminating hypoxic deep-seated tumors in HCC models. Such models may also elucidate socioeconomic mediators—limited healthcare access correlates with delayed interventions and reduced treatment adherence, potentially exacerbating disparities in outcomes.

Future research should focus on: multicenter phase II clinical trials (stratifying patients by tumor size to evaluate TACE efficacy with PFS as the primary endpoint) to elucidate STS-specific immunomicroenvironmental characteristics, particularly in distinct subtypes (25); mechanistic studies integrating perfusion MRI with serial biopsies to analyze spatial heterogeneity of TACE-induced necrosis and dynamic changes in the immunomicroenvironment (e.g., PD-L1/tumor mutational burden) for developing more effective combination therapies, such as integrating interventional therapy with ICIs, chemotherapy, or radiotherapy; and real-world registry studies (using standardized metrics like RECIST 1.1, MSTS-93, and EQ-5D) to validate the comparative efficacy of TACE versus systemic therapies, while exploring combined local modalities like radiofrequency or microwave ablation for recurrent UPS to improve local control rates (13).

## 4 Conclusion

This study reports the efficacy of two TACE procedures in a patient with recurrent UPS of the right calf. Superselective embolization of tumor-feeding arteries combined with chemotherapeutic infusion achieved significant tumor shrinkage, necrosis, and long-term disease stabilization. The case highlights TACE as a viable locoregional treatment for hypervascular recurrent UPS, particularly in cases refractory to surgery or conventional therapies.

## Data Availability

The original contributions presented in the study are included in the article/supplementary material. Further inquiries can be directed to the corresponding author.
